# Current Research Trends in the Application of In Vitro Three-Dimensional Models of Liver Cells

**DOI:** 10.3390/pharmaceutics15010054

**Published:** 2022-12-24

**Authors:** Chawon Yun, Sou Hyun Kim, Young-Suk Jung

**Affiliations:** Department of Pharmacy, College of Pharmacy, Research Institute for Drug Development, Pusan National University, Busan 46241, Republic of Korea

**Keywords:** 3D cell culture, hydrogel scaffolds, spheroid, organoid, organ-on-chips

## Abstract

The liver produces and stores various nutrients that are necessary for the body and serves as a chemical plant, metabolizing carbohydrates, fats, hormones, vitamins, and minerals. It is also a vital organ for detoxifying drugs and exogenous harmful substances. Culturing liver cells in vitro under three-dimensional (3D) conditions is considered a primary mechanism for liver tissue engineering. The 3D cell culture system is designed to allow cells to interact in an artificially created environment and has the advantage of mimicking the physiological characteristics of cells in vivo. This system facilitates contact between the cells and the extracellular matrix. Several technically different approaches have been proposed, including bioreactors, chips, and plate-based systems in fluid or static media composed of chemically diverse materials. Compared to conventional two-dimensional monolayer culture in vitro models, the ability to predict the function of the tissues, including the drug metabolism and chemical toxicity, has been enhanced by developing three-dimensional liver culture models. This review discussed the methodology of 3D cell cultures and summarized the advantages of an in vitro liver platform using 3D culture technology.

## 1. Introduction

Efficient drug discovery and development relies on a model system that accurately predicts the human drug response and the drug’s toxicity before entering the clinical stage. However, differences in species, especially in the expression and specificity of liver enzymes, dramatically reduce the prediction accuracy, which is a considerable obstacle for developing new drugs. Isolated primary human hepatocytes cultured in two-dimensional (2D) configurations have long been used in liver biology and in the research on their function, drug metabolism, and toxicity in order to overcome this problem. However, the 2D culture system has limitations as it differs from in vivo conditions. Seeding liver cells in the laboratory on a plastic culture plate coated with specific compounds results in a rapid loss of liver function mediated by bursts of various microRNAs that inhibit liver gene expression 30 min after their exposure to the substrate [1,2]. Over the past 10 years, it has been clear that the 2D culture of hepatocytes has important limitations; this system is, therefore, not the best for reproducing the biological and drug reactions in vivo.

Three-dimensional (3D) cell technology is a field that has been steadily developing since the 1990s because of its excellent bioreactivity compared to two-dimensional cell culture systems. A cell culture in 3D refers to technology that cultivates cells in a 3D space composed of an extracellular matrix (ECM) and various growth factors. Three-dimensional cell culture systems provide a bio-like environment by supplying cells with nutrients and other factors that are necessary for the cells to grow. This system enables 3D contact between cells, intercellular interactions, and communication between cells and extracellular substrates, resulting in signal transmission through the diffusion of cell secretions that do not appear in 2D cultures.

A 3D culture model can mimic the microenvironment around the tissues, where cells can proliferate and differentiate [3]. In particular, cells cultured in 3D showed different reactions to drugs than cells cultured in 2D [4]. As summarized in Table 1, the 3D cell culture model has been reported to be more bioreactive than the 2D cell culture model in terms of cell shape, cell differentiation, and the drug metabolism (Table 1). 

Compared to cells grown in 3D, cells grown in 2D cannot maintain their standard form and, hence, are more sensitive to drugs [6]. Another difference is in the surface receptors of the cell [4]. Drugs often target specific receptors on the cell surface. Therefore, differences in the surface receptors’ structures and spatial arrangements can affect the binding of drugs to receptors, inducing different reactions. In addition, most cells cultured in 2D are in the same cell stage, whereas 3D cells often progress through different cell stages [10], which is very similar to those in vivo. Therefore, there have been many restrictions on using 2D models, as tissue cells in vitro often present inaccurate results. In this review, we aimed to understand the technology of 3D cell cultures and to summarize the research on the liver metabolism using 3D culture technology.

## 2. Three-Dimensional Cell Culture Technology

Over the past decade, the research on 3D cultures has raised the need to increase the efficiency of research and development, primarily in the pharmaceutical field, in order to improve productivity. These requirements and efforts have accelerated the use of 3D cell culture technology in developing early drugs. Using better cell models, such as inducible pluripotent stem cells (iPSCs) and primary cells, 3D technology enables a better prediction of the human efficacy and toxicity of drugs before they enter clinical trials [11], allowing the prediction of the consumption rate of new medicines that are under development. In addition, the establishment of 3D cell cultures and coculture models has the advantage of enabling drug safety and efficacy evaluations to be set in a more in vivo-like context than that of conventional 2D cell cultures and often eliminates the differences in species outcomes.

**Figure 1:**
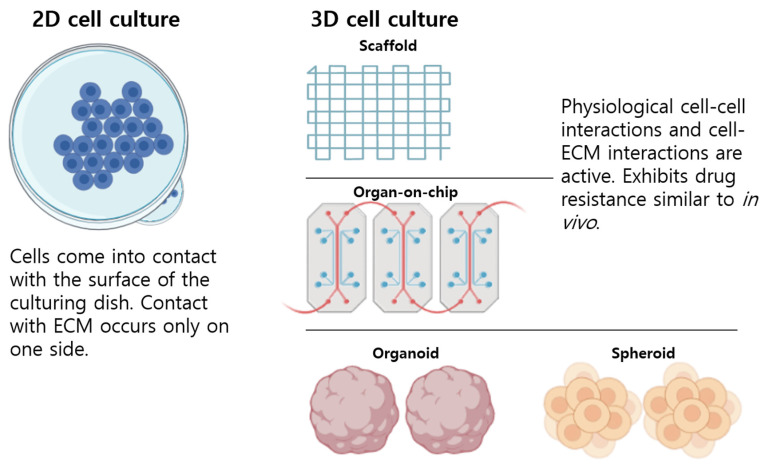
A basic comparison of two- and three-dimensional (2D and 3D) cell culture systems. Improved forms of in vitro cell culture models are similar to in vivo conditions. Three-dimensional cell cultures mimic the tissue physiology of multicellular organisms.

In this review, we explained the methods used in 3D cell cultures. These are largely divided into (1) a scaffold-based system in which cells exist in 3D conditions in an extracellular matrix and (2) a scaffold-free system wherein cells grow together without an extracellular matrix (Figure 1).

### 2.1. Hydrogel Scaffolds

Hydrogel scaffolds are the most widely used technology in 3D cell cultures and use advanced 3D printing techniques [12]. The scaffold provides an environment in which cells can stably adhere and grow by placing hydrogels in a 3D frame made using 3D printing techniques.

Cells have been encapsulated in microporous, nanofibrous, and hydrogel scaffolds to overcome the limitations of 2D cell cultures and to grow cells more effectively in the laboratory [13]. The microporous system has the advantage of conveniently suturing cells, which effectively serves as a 2D support with curvature because the particle size of the material used is larger than that of the cells [14], but it is associated with some disadvantages, especially uncontrollable porous structures [15]. Nanofiber scaffolds can better create extracellular structures formed by fibrous proteins [16]. However, their limited control over the pore structure is a significant disadvantage of nanofiber scaffold technology. Hydrogels have been developed to overcome these limitations. The synthesized cell growth environment can achieve cell survival only if the cells can modify the surrounding microenvironment. In general, acceptable hydrogels, synthetic polymers, provide a good 3D environment for cell cultures but have the disadvantage of not being able to activate integrin and other surface receptors. However, hydrogels formed from naturally induced polymers have been shown to promote the activity of numerous binding sites and growth factors, which is coordinated by additional cell responses through signal cascades combined with cell surface receptors [13].

Scaffold-based culture technology can directly provide physical support, such as that of an extracellular matrix, to allow cells to gather, proliferate, and move to where they are needed. In this system, cells are transplanted into a matrix within the scaffold. Hence, the scaffold material determines the properties of the cells. The purpose of the scaffold is to enable the function of most native cells within the ECM. Three-dimensional scaffolds are generally biocompatible and allow the appearance and function of cellular structures to be similar to those of their original environment [17]. The key to the design of the scaffold is that scaffold must determine the organization’s interests, and the decision determines the composition of the scaffold. Therefore, the larger or more complex the size, the more difficult it is to extract cells for analysis [18].

Biomaterials for hydrogel formation are classified as natural (derived from plants or animals) and synthetic (artificially produced). Natural biomaterials consist of peptides, such as gelatin, or polysaccharides, such as alginate, cellulose, chitosan, and hyaluronic acid.

Alginate is a naturally occurring biocompatible polysaccharide that is not found in mammals [19]. It is rich and inexpensive but has a low cell adhesion affinity. Therefore, it is often combined with other substances to increase the possibility of adjustments to its properties and to act as support for implanted cells. Alginate is water soluble with a high water-holding capacity. Materials generated using alginate are thermally stable, and their stiffness can be easily adjusted [20]. This system is used for neck squamous cell carcinoma and hepatocellular carcinoma responses [21].

Cellulose is a natural material extracted from the plant cell wall and has the advantage of low manufacturing costs because it is abundant and easily produced in nature. In a cellulose scaffold, cells can be attached to cellulose through a hydrophobic hydroxyl moiety and a specialized cellulose-binding domain [22]. Cellulose is physiologically active and has biomechanical properties suitable for liver transplantation, so it has been widely used for culturing liver cells. Tests on the ability of nanofibril cellulose hydrogels to induce the formation and differentiation of the rotating ellipses of HepaRG and HepG2 show that, even if only cellulose hydrogels are injected without adding other bioactive ingredients, the cells form a 3D scaffold and promote the formation and differentiation of the hepatocytes. One disadvantage of cellulose is that it cannot be decomposed naturally and can be considered a permanent structure. However, this may also provide long-term structural support that may benefit the liver tissue when combined with the delivery of cells capable of performing the function of liver cells [22,23].

Chitosan is generated from crustacean shells and has a linear cationic polymer. Chitosan is used to generate polyelectrolyte complexes with anionic polymers [24]. It is also used as a blood clotting inhibitor because it is highly resistant to cell scaffolding [25]. Chitosan often binds to other substances or functional molecules when it acts as a cell transplant material because of its lack of cell-binding domains and its poor bioactivity with the cells. For example, lactose moieties can be bonded to chitosan to promote cell adhesion and to adjust the mechanical properties of its materials to improve its cell adhesion, biocompatibility, and mechanical stability in 3D scaffolds used for liver cells under culture [26]. Chitosan scaffolding used in the 3D culture system of cancer cells has also been reported. When human breast MCF-7 cancer cells were cultured in chitosan, cell adhesion and proliferation were superior to 2D cultures. Liver, lung, and prostate cancer cells are also available to be cultured in chitosan scaffolds [27].

Gelatin maintains the composition of collagen. It is biodegradable, affordable, and can be easily produced alone or in 3D scaffolds with other biomaterials. Gelatin can be combined with methacrylic anhydride to synthesize gelatin methacryloyl (GelMA). GelMA is biocompatible and has mechanical properties that can be decomposed and adjusted by matrix metalloproteases (MMPs) [28]. Gelatin material scaffolds are used in the coculture systems of cancer cells and stromal cells. They are mainly used in breast, liver, lung, and pancreatic cancer cell cultures. [29]

Hyaluronic acid (HA) is a natural material widely used for cell transplantation owing to its biocompatibility and adjustable properties [30]. It is abundant in increased cell proliferation environments such as embryonic development, wound recovery, and regeneration. In addition, the backbone of HA includes carboxyl and hydroxyl groups that can function as different molecules for the addition of bridging or survival promoters in order to adjust hydrogel stiffness, and many researchers have demonstrated the efficacy of HA in transferring different cell types to tissues [31]. HA has an affinity for the CD44 surface receptor, which affects the cell function. As CD44 is a major surface marker of stem cells, HA affects the stemness, tumorigenesis, and drug resistance of many cancer cells [32]. For example, HA can be used to culture various cancer cells, such as glioblastoma, lung cancer, gastric cancer, prostate cancer, breast and liver cancer, osteosarcoma, and endometrial adenocarcinoma. A consideration for HA use is that low-molecular-weight HA production has been reported to increase liver fibrosis in human and mouse models. It has been reported that this is due to fibrosis phenotypes promoted through TLR4 and CD44 signals, through proliferation, and through the invasion of hepatic cells. This result suggests that, although there are certainly many advantages in using HA, further work is essential to characterize the host’s response to HA [33].

Synthetic materials are also widely used in the manufacturing of scaffolds for cell transplantation, and the advantages of synthetic polymers are that they are readily available, they have better mechanical properties, and their decomposition can be mechanically adjusted [34]. However, owing to the disadvantage of their low binding affinity to cells, biomolecules are usually added. Also, the results may not be suitable for living things. Many decomposition products are still acidic and can create unfavorable microenvironments for cell transplantation. They are widely used in the regeneration of tissues, such as blood vessel, nerve, lung, and liver tissues. 

Poly(ε-caprolactone) (PCL) is characteristically soft and flexible. PCL is known to have an advantage over other synthetic or natural biomaterials in its ability to easily control pore size and fiber orientation during manufacturing [35]. When PCL and chitosan are mixed and used, they are combined into nanofiber scaffolds, which could promote the infiltration of mouse liver epithelial cells by controlling the pore size of the scaffold [36].

Poly(lactic-co-glycolic) acid (PLGA) is synthesized by combining PLA and polyglycolic acid (PGA), which can often be combined with natural materials to form a hybrid scaffold for tissue cultures [37]. That is, when PLGA is coated with collagen, a scaffold filled with softer 3D collagen hydrogels may be formed [38]. If other encapsulated cells are transplanted and cultured, they can survive for a long time.

Polylactic acid (PLA) and its derivative, poly-L-lactic acid (PLLA), are known to be biodegradable. These copolymers can be artificially adjusted to have a wide range of mechanical properties [38]. When decomposed, PLA and PLLA form lactic acid monomers metabolized by carbon dioxide and water. In this case, they can be formulated to be easily used. For example, a popular use of PLA and PLLA, for example, is their use in beauty implants in clinical cases.

### 2.2. Organ-On-Chips (Microchips)

Microchips are organ-on-chips which mimic a living body and provide a biological environment similar to the human body by placing and cultivating organ cells, such as blood vessel, lung, and liver cells, on a small chip [39]. A microchip is a particular type of microfluidic chip that mimics the function of human organs within a test tube, including the microenvironment of cells, coculture of cells, and interactions between cells. The organ-on-a-chip model can more accurately adjust the environmental conditions related to growth and function, depending on the type of cell being cultured [40,41]. Flow cultures can control the nutrient supply, remove accumulated cell waste or secondary metabolites from culture media, and control oxygen levels. Cell reconstruction also ensures the barrier function of the cell layer and controls cell movement within the test tubes. Cells in the organ-on-a-chip model can be used to regenerate many functions that are difficult to achieve in conventional in vitro models, such as the reconstruction of tissue barrier functions, the culture of parenchymal tissues, and the integration of multiple organ functions.

Testing the drug toxicity is very important for identifying the problems the drug may cause after marketing. The preclinical toxicity testing of candidate drugs is essential. However, owing to the genetic differences between humans and other species, the results obtained from animal models may not be reliable [40,42]. Therefore, many toxicity screening methods have been developed to improve the accuracy of clinical trials when testing drugs in humans. Among the many established in vitro models, microchips have been found to provide excellent results.

Microchips are advantageous as they allow for the testing of the drug toxicity by forming multiple organs on a single chip. For example, in 2017, Chang et al. reported a model in which the liver and kidneys were formed on a single chip in order to test the toxicity of aristolochic acids in the human kidneys [43]. Currently, the number of laboratories using “Organ-on-Chips” for new drug development is increasing. For example, the Wyss Institute reported that it developed and used a “lung-on-chip” similar to the human lung [44]. In addition, a heart-on-a-chip with an environment similar to the human heart was developed and used. In addition, chips applying various human cells, such as kidney, intestine, muscle, bone, and skin cells, are being developed. The reason for developing microchip technology is that it can reduce the time required for animal testing and quickly generate new data. While animal models continue to be used, they do not fully represent human biology and cannot replace humans in testing [45].

**Figure 2:**
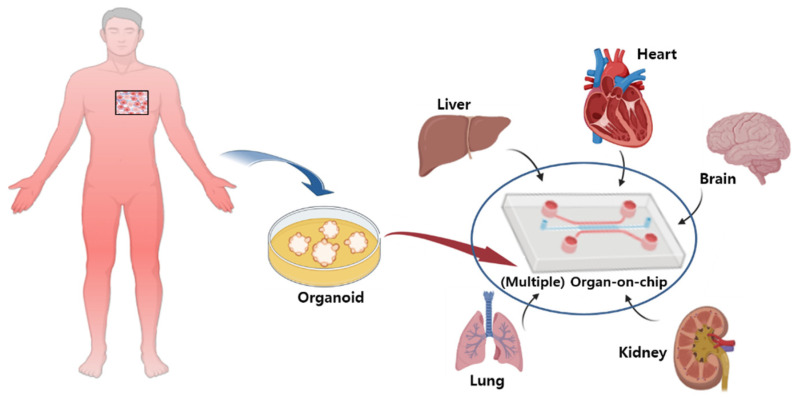
Multi “organ-on-chip” or microchip platform model for disease research. The organ-on-chip model mimics the physicochemical microenvironment of tissues in the human body. It is a microfluidic cell culture system with well-controlled conditions applied to study various diseases.

The advantage of the “Organ-on-Chip” is that it compensates for the shortcomings of previous preclinical experiments. It might anticipate the health problems of clinical trial participants and accurately extract the human response data that may appear in human trials. In addition, it is possible to examine the complex reactions in the human body by applying several chips simultaneously [46] (Figure 2).

### 2.3. Organoids and Spheroids

**Figure 3:**
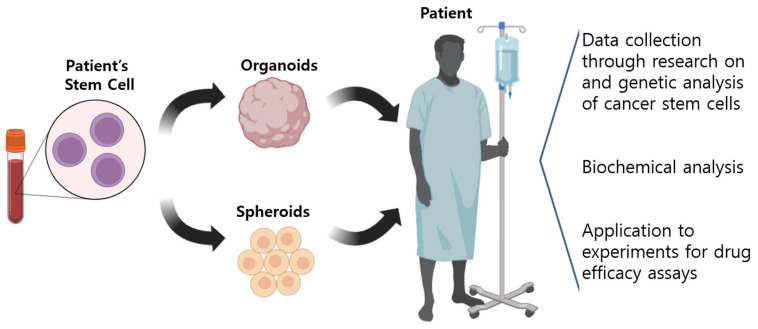
Clinical model for isolating spheroids and organoids from patients. Three-dimensional in vitro models, such as tumor spheres and organoids, can be used to evaluate drug responses at individual patient level by providing new opportunities to build and apply three-dimensional in vitro human tumor models used for oncology or immunotherapy studies and various drug screenings.

The cell culture technology that has recently attracted the most attention is 3D organoids, or mini-organs made by culturing or recombining organ cells [47]. Initially, stem cells obtained from a rat’s intestines were cultured to allow cells identical to the rat’s rectum to grow in 3D. One of the most significant advantages of such organoids is that their structural characteristics are similar to those of human organs. In particular, patient-tailored experiments are possible when culturing cancer cell organoids because they can more accurately predict how patients will react to certain anticancer drugs (Figure 3).

Among 3D models, organoids and spheroids are the most interesting and promising models because they can recheck pathophysiological problems along with the heterogeneity of human cancers and can reduce the gap between existing in vitro experiments and animal models using 2D models [48]. These 3D systems are also powerful tools for studying cancer biology, as they have the advantage of modeling the dynamic evolution of neoplasm-related diseases from the early stages to metastasis and of modeling the interactions with microenvironments [49]. Organoids and spheroids have recently been used to discover new drugs and patient-specific medical cases (Table 2). These 3D models can potentially enhance the robustness and reliability of preclinical data and dramatically reduce the need for animal testing.

#### 2.3.1. Organoids

The organoid culture model, emerging as a hot issue in 3D cell culture modeling, resembles the structure and function of the organs, such as the stomach and liver. It is used for disease research and drug development by enabling bioresponsive disease models to be implemented outside the body [54]. Many researchers have concluded that organoids are the final stage of 3D development as the final purpose of the 3D cell culture model is to create a highly biocompatible model in vitro.

Organoids are clusters of stem cells or precursor cells bound together in extracellular environments, such as matrices and collagen, and they are growing into the microscopic dimensions used in 3D research [55]. Organoids have been used for applications in medical research and have been optimized for disease modeling and drug discovery, improving the studies of organ development. According to PubMed, the number of papers on organoids worldwide was 36 in 2010, increasing to over one thousand in 2019 and growing steadily [56].

Organoids have been used in disease research and drug development by implementing disease models with excellent responsiveness in vivo and in vitro. According to Lancaster and Knoblich’s definition [57], three factors are needed to grow an organoid. First, it must have at least one cell species of the organ it is trying to imitate. Second, it must express the specialized functions of the organ it is trying to imitate, and third, it must represent a form similar to the organ it is trying to imitate [58]. Therefore, organoids cannot be produced solely by forming cell masses.

#### 2.3.2. Spheroids

Spheroids are simple clusters of broad-ranging cells, such as tumor tissues, embryo bodies, hepatocytes, nervous tissues, and mammary glands [49]. Spheroids do not require scaffolding to form 3D clusters [59] but simply stick to each other. However, they cannot self-assemble or regenerate and, thus, are not as advanced as organoids.

Spheroids have been of great help in cancer research. Scientists have gained much knowledge by using spheroids to understand the tumor microenvironment, allowing them to predict drug efficacies against cancer. Early spheroids were mainly developed in the 1970s to study the effects of radiation therapy on human tumor cells. Since then, they have been used in stem cell research to develop embryos from iPSCs [60].

Generally, a spheroid contains cells exposed to the surface and a layer of cells buried in the sphere. Spheroids generally contain proliferative, nonproliferative, and necrotizing cells, and they have the disadvantage of being well oxygenated or hypoxic [61]. Understanding the formation of tumor cells is one of the most important goals in cancer research. Spheroids imitate the contents of solid tumors much more accurately than 2D cell cultures [62], facilitating the study of the physiological changes that distinguish tumor cells from healthy cells. In particular, using various cell types, tumor spheroids provide deeper knowledge and information about the tumor microenvironment, increasing our understanding of how tumor cells absorb nutrients and multiply. Spheroids are also useful in developing clinical treatments for monitor the cell responses to drugs [51]. In other words, it is the ideal method to use for drug screening and efficacy testing before clinical applications. Spheroids have been applied in many studies of tumor biology, offering lower costs, reproducibility, high throughput, ease of data integration, and connectivity with advanced imaging technologies, and they are less labor-intensive compared to animal models.

The greatest advantage of spheroids is that they are more viable and can express various genes compared to 2D monolayer-cultured tissues. In particular, the genes function similarly to those of natural tissues because they contain the unique gene transcription factors present in human tissues. Therefore, expressing the characteristics of a specific tissue effectively and stably is very valuable in tumor research [63]. Recently, spheroids have been applied in studies on the carcinogenesis mechanisms and cell-necrosis-forming processes by using gene function analyses, evaluating the tumor therapeutic efficacy, evaluating the differentiation processes of tissues that differentiate into different tissues, and using tissue engineering related to organ regeneration.

## 3. Advantages of 3D Cultures in Studies on the Liver Metabolism

The liver plays an important role in preserving and controlling the body’s homeostasis of the lipid and glucose levels as well as the energy balance. Normal liver function can be disrupted by various causes such as viruses, drugs, and poisons, which can ultimately cause fibrosis and cirrhosis. Chronic liver disease and cirrhosis are extensive and incurable conditions that lead to lifelong damage and eventual death. However, owing to the disparity in the supply and demand of livers for transplants, there is a significant lack of options for patients to take the initiative with. Accordingly, it is necessary to weaken the progression of the disease and to rebuild liver function by developing new liver regeneration promotion strategies, and many studies are being conducted on drug development together with liver-material science.

The liver metabolism is an important process for the efficacy of drugs. Changes in drugs caused by microsomes and nonmicrosomal enzymes in the liver are important factors in determining the efficacy, toxicity, and pharmacological effects of drugs [64]. In particular, cytochrome P450 (CYP450), a family of heme-containing microsomal enzymes, catalyzes various reactions, such as oxygenation, dehydrogenation, and reduction, which are key processes for the biological conversion in living organisms [65]. Cells grown in a 3D culture express an increased amount of the CYP450 enzyme. Hence, their drug metabolism occurs more quickly than in that of cells in 2D cultures. In addition, 3D cultures possess not only a higher drug metabolism than 2D cultures, but they can also reproduce a drug resistance similar to that observed in vivo [66]. In many cases, drugs that were effective in 2D cultures were ineffective or very weak at the beginning of clinical trials, making their use impossible. Three-dimensional cultures show similar results to the in vivo metabolism and can indicate the toxicity and effects of drugs similar to those in vivo without animal experiments [67]. Therefore, 3D cultures can provide data more relevant to clinical trials, reducing the failure rate. This further emphasizes the importance of 3D cultures for selecting target substances before running expensive clinical trials. For example, in the case of liver cancer, a drug resistance that did not appear in 2D cultures was shown when a drug that prevented DNA synthesis was administered for about 6 days to a 3D culture of liver cancer cells. Because cancers exhibit drug resistance, 3D cultures show more bio-like results than 2D cultures [68,69]. In addition, cancer cells are in a hypoxic state and have different nutrient metabolisms [70]. In this case, the 3D model can naturally induce hypoxia (hypoxia) and signal transmission and interaction between the cells; therefore, it is also relevant in testing anticancer treatment effects.

Thus far, several different types of 3D cultural models have been established, as discussed in this review. However, liver metabolism research has been hindered by the limited ability of primary liver cells to expand in vitro while maintaining their metabolic function. Therefore, 3D culture technology is being developed under optimized conditions for the testing of metabolic mechanisms to compensate for this [71,72].

### 3.1. Liver Study with Scaffolds

3D hepatocellular cultures using biomaterials are becoming an increasingly promising option in culturing and delivering cells to support the survival and liver function after transplantation [33]. Current cell transplant levels often have difficulty integrating into the liver owing to low post-transplantation cell survival rates and the loss or lack of a substantial ECM and the vascular structure of the liver. As a result, researchers are seeking ways to support transplanted cells and to deliver cells within biomaterial structures to provide a foothold for post-transplantation regeneration.

An endogenous ECM must be produced to be used as a biomaterial for hepatocytes. Also, 3D structures must be supported, and the key features of stiffness, protein composition, and angiogenesis characteristics must be at least partially reproducible [52]. Using these biomaterials allows cells to be transplanted directly into the liver without going through the hepatic portal vein, resulting in more cells being transferred to the liver regeneration site. For this application to be successful, basic liver functions such as urea cycle maintenance, albumin synthesis, drug metabolism, and xenobiotic detoxification must be maintained in a 3D environment [73].

In addition to the properties of the materials, matrix stiffness is also known to be able to regulate the intracellular signaling pathways that are important for spreading, intrinsic cellular contractility, cell migration (durotaxis), and cell proliferation [74]. Rigidity directly affects nonsubstantial interactions, cellular adhesion and cohesion, cellular motility, and responsiveness to the growth factors of the skeleton. In particular, as liver disease progresses and tissues become fibrous in the liver, rigidity increases, so it should be adjusted to suit healthy liver rigidity or various pathological conditions for in vitro research. In general, the hepatocellular function decreases as stiffness increases, which is also a task to overcome [75]. For example, increasing the stiffness of the polyelectrolyte multilayer reduces the albumin production of hepatocytes but increases the stiffness of the polyacrylamide gel, thereby improving the hepatocyte function.

Alginate is abundant and inexpensive but has low cell adhesion, so it often combines with other substances to increase the possibility of the adjustment of its properties and supports implanted liver cells. The Arg-Gly-Asp (RGD)-modified chitosan–alginate polyelectrolyte composite fiber scaffold has been demonstrated to support the delivery and survival of human mesenchymal stem cells in the hepatic rat model. In other words, when it is transplanted into the liver using a scaffold, cell survival increases after 14 days, human albumin is produced, and differentiated liver phenotypes are well maintained [76]. This research demonstrates that the albumin secretion and urea synthesis of primary human liver cells are maintained in microcapsule systems. This is done by fabricating microcapsules based on galactosylated alginate to mimic the liver microenvironment using an alginate-based 3D culture system [76].

Cellulose is physiologically active and has biomechanical properties suitable for liver transplantation, so it has been widely used for culturing liver cells. It has been found that the injection of cellulose hydrogels forms a 3D scaffold and promotes the formation and differentiation of hepatocyte spheroids [77]. Meanwhile, several results have been reported that mixing collagen with other compounds can increase the stability of cells growing under 3D conditions. For example, when collagen is mixed with PLA, hepatocytes grow in nanofibers by forming cell aggregates, providing mechanical support for cell growth and differentiation and exhibiting stable functional expression, including superior cell retention, cell activity, and albumin synthesis [78]. Also, when hepatocytes were cultured in cellulose and chitosan complex hydrogels, the cells exhibited the enhanced secretion of glutamate-oxaloacetate transaminase and glucose [79].

Chitosan, a partially deacetylated derivative of chitin, is biocompatible and biodegradable. It is used in various ways as medical and pharmaceutical materials. Chitosan is used in hepatocellular cultures, as its structure is similar to glycosaminoglycans (GAGs), a component of the liver extracellular matrix [80]. GAGs are essential components of the adhesion molecules and matrix glycoproteins required in many cells. In a recent experiment using a 3D liver support for in vitro cultures, the chitosan–gelatin scaffold was found to be inexpensive, easy to manufacture, and noncytotoxic [81]. It was also found that the porous structure of chitosan–gelatin is similar to the ECM, which promotes hepatocellular adhesion and proliferation. As a result of growing HepG2 cells in hydrogel using chitosan, the cells were self-aggregated, spherical, and showed a higher liver-specific function. In other words, albumin secretion and urea synthesis were increased [82].

Gelatin is being used successfully in the cultivation of liver cells. Gelatin was mainly used as a 3D porous scaffold to support in vitro hepatocyte cultures, as it increases hepatocellular viability and the culture’s ability to use a complex glutaraldehyde–chitosan–gelatin 3D scaffold [83]. In addition, when gelatin is cultured in combination with galactose or laminin, the differentiation and survival of liver cells increases, and the expression of liver-cell-specific genes and markers such as albumin increases [84]. Gelatin can also be combined with methacrylic acid to synthesize gelatin methacryloyl (GelMA). GelMA is biocompatible and has mechanical properties that can be decomposed and adjusted by MMPs. It is used not only for 3D printed bioink in adult hepatocellular cultures, but also for making 3D lobular-like tissues with hepatocytes and fibroblasts [33]. In particular, mixing gelatin with nanofibrillar cellulose and HA promotes the liver differentiation of HepaRG liver precursor cells [85].

Hyaluronic acid (HA) is another important component of the extracellular matrix. HA is involved in cell proliferation and expansion regulation [86]. The immature and mature hepatocytes of fetal and adult liver cells express CD44 as a surface receptor for hyaluronic acid [87]. HA hydrogels, and their derivatives, are synthesized using the characteristics of these hepatocytes in order to have more adhesion to the surrounding hepatocytes. Recently, cell therapy for liver disease using human biliary stem cells (hBTSCs) has been recognized to have a low transplantation efficiency. However, it is known that coating hBTSCs with HA increases cell survival and proliferation [88]. This is owed to the fact that the HA-coating promotes HA’s biological properties, which lies with its ability to maintain the essential cell adhesion molecules required to enhance the cell–cell adhesion and cell–cell interaction. It was also reported that the hiPS-hepatocytes in a material made by homogenizing HA with PEG or collagen I show a significantly increased survival capacity and high albumin production [89,90]. As such, many studies have been conducted recently to promote the function of liver cells by using HA alone or in combination with other materials.

Synthetic substances have also been widely used for cell transplants to the liver. For liver regeneration, PCL can be manufactured in various scaffold types, such as nanofibers and 3D porous structures [33]. It was possible to promote the mouse liver epithelial cell infiltration by using a material mixed with PCL and chitosan [36]. In addition, it has been reported that combining an injected natural decellularized ECM with PCL and with HepG2 cells treated as factors promoting ECM secretion can better support the in vitro HepG2 survival and expression of liver-specific genes than scaffolds without an ECM deposition [91]. In addition, it has been shown that using PLA combined with other synthetic materials, such as PCL, combined with natural substances, such as collagen, allows the promotion of hepatocellular differentiation from human mesenchymal stem cells.

PLGA may control the environment for liver regeneration and provide various mechanical properties of liver tissue [33]. It was confirmed that, when the primary liver cells of encapsulated mice were transplanted by mixing PLGA with natural substances to form a hybrid scaffold for hepatocellular cultures, collagen was dispersed in the 3D space after 10 days of culturing cells [92]. As a result, hepatocyte aggregation and albumin secretion occurred in this structure. In addition, a direct comparison of the addition of fibronectin or collagen type I in the 3D nanofiber PLGA scaffold for primary human hepatocellular cultures confirmed that the addition of collagen type I was the best condition for the testing of the liver-specific function [93].

PLA and PLLA also have had various attempts to support the viability and function of liver cells [33,94]. It has been reported that hepatocytes grow well when fetal liver cells are cultured on a 3D printed PLLA scaffold. The binding of the scaffold with cytokine oncostatin M stimulates the maturation of the liver parenchyma cells in the culture of the liver cells [95]. After combining PLA and gelatin and adding FGF to the mesenteric membrane of a mouse with a liver resection, angiogenesis and the hepatocellular survival and function were confirmed to increase the growth of all indicators. Meanwhile, as manufacturing technologies such as 3D bioprinting continue to develop, various methods are being studied to customize synthetic-based scaffolds for hepatocellular cultures and transplantation, and research on these synthetic materials is also steadily developing.

### 3.2. Liver Organ-On-Chip Studies

The liver plays an important role in the metabolism and detoxification, glycogen storage, and synthesis of secretory proteins in the body and is one of the largest organs, and it is the main target organ of drug toxicity [96,97,98]. Drug-induced liver injuries (DILIs) can quickly cause acute and chronic liver disease followed by drug prohibition. Therefore, the toxicity and stability tests of drugs are major issues in developing new drugs. However, many drug candidates have not been approved because of their association with drug-induced liver damage [99,100]. Therefore, the liver is an important target organ for the 3D application in drug development. To this end, liver-on-a-chip is a state-of-the-art technology that can confirm the drug hepatotoxicity [40]. A unique advantage of a microchip/organ-on-a-chip is that it can integrate the metabolic and toxic processes of drugs into a single device, making it more convenient to use when evaluating the toxicity of drug metabolites [40].

Gaining information on drugs with respect to the boundary between the drugs and their toxicity in a 2D cell culture system is still a challenge that can be overcome with the organ-on-a-chip model. This allows the laboratory composition of the structural complexity of body tissues to provide readings of the interactions of different tissue types as well as liver tissues, providing various results for the physiology of the chemicals of interest.

Reproducing the liver in vitro is still a big challenge as different types of cells are mixed to form complex cell structures or are randomly seeded under culture conditions that make it impossible to manipulate the intercellular interactions. This makes it difficult for even the culture system to represent complex liver situations accurately. However, rapid advances in microprocessing and microfluidic technologies have provided a promising approach to building microsized functional liver structures on chips. In addition, microfluidic devices have many advantages over conventional cultures, which can efficiently perform concentration gradients, control cell space distribution as planned, and provide the desired flow environment. Based on the classification method reported by Deng in 2019, the current general method of building a liver-on-a-chip and its advantages and disadvantages are summarized below [101].

A liver-on-a-chip with matrix-less 3D spheroid aggregates hepatocytes into 3D spheroids [101,102]. This is another conventional method and a promising in vitro model for liver metabolism and cytotoxicity studies [102]. In this method, liver cells are spontaneously deformed according to the material’s surface to form a spheroid, which is a rotational ellipse, and the mass production of a uniform-sized spheroid is easy. In addition, the survival rate of spheroids is high, and albumin secretion is active. In addition, it has been found that the metabolic activity related to the long function of the cells increases significantly. In addition, experiments using the hepatocytes in humans have shown that liver-specific protein synthesis, CYP450 activity, and phase 2 and phase 3 drug-metabolizing-enzyme gene expression and activity are maintained [103]. The hanging drop method, an early stage of spheroid formation, is a scaffold-free system and can form microstructures without force or synthetic material in addition to gravity [104]. Despite these advantages, the hanging drop method does not have a standardized protocol, so the results appear differently depending on the user. Therefore, the establishment of standardized techniques that can maintain certain conditions is a challenge to overcome.

A liver-on-a-chip with a matrix-dependent 3D system encapsulates cells within a 3D matrix, such as hydrogels, BMEs, and collagen, which replicates the supporting functions of the extracellular matrix [101]. In liver tissue engineering, scaffolds such as an ECM are needed to promote cell adhesion, support cell growth, and improve the cell–matrix interactions, and various ECM components have been applied to the liver to improve the liver function in pharmaceutical and cytotoxic applications. To this end, an ECM was used in the liver chip to maintain and imitate the unique microenvironment of the liver. As a result, cultured hepatocytes on coated chips exhibited both cell–cell and cell-ECM interactions and maintain hepatocellular synthesis and metabolic function [105]. Hepatocytes were also introduced into the liver chip as a pre-gel ECM component solution, showing that hepatocytes on the chip exhibit higher albumin and urea secretion as well as higher collagen secretion under perfusion. As described in Section 2, hydrogels have a number of essential important functions that mimic the basic mechanical and structural signals that promote cell attachment, proliferation, and differentiation. In this way, hepG2 cells or iPSC-derived hepatocytes (h-iPS-HEP) were encapsulated in the HA-PEG hydrogels of perfusion devices and were implemented as a liver-on-a-chip. The encapsulated HepG2 cells formed high-survival spheroids and showed high albumin and urea secretion. In addition, h-iPS-HEP was transferred and grown in 3D within HA-PEG hydrogels transformed into RGD peptides and showed increased viability and higher albumin secretion compared to other hydrogels [106]. However, the results can vary depending on the stability and rigidity of the matrix used and the interval of arrangement, so this part needs to be constant.

A liver-on-a-chip based on 3D bioprinting can generate anatomically accurate liver anatomy, including the specific spatial structures and vascular networks of the liver, and the unique aspects of this technique are becoming increasingly popular as tools for manufacturing in vitro liver models in order to study liver disease and to screen drugs [101]. The HepG2/C3A spheroids cultured in this manner were incubated in a bioreactor chamber for 30 days, and the printed cell-rotating ellipsoid exhibited liver-specific functions, including the secretion of albumin, antitrypsin, transferrin, and ceruloplasmin [107]. This result demonstrated a considerable improvement in the liver function due to the formation of bile systems using chips that have become effective potential candidates for drug discovery. However, as there is a difference in the results according to the accuracy of printing, the results of this system also differ depending on the degree of development of 3D bioprinting.

A liver-on-a-chip with a layer-by-layer deposition method imitates the unique structural features of liver sinusoidal waves, a functional repeating microvascular unit formed by sinus walls consisting of endothelial cells connected to the hepatic portal vein and hepatic artery [101]. Owing to advances in microprocessing and microfluidic technologies, this method has become a promising platform for summarizing the important functions of hepatic sinusoidal waves. The simultaneous injection of HepG2 cells into the chip with collagen-containing HUVEC confirmed that the two collagen layers formed a clear boundary using the laminar flow of the system and eventually self-assembled into a single layer [108]. This method can easily control the location of the cell layer in order to mimic the distribution of liver cells and form tightly connected endothelial cells for perfusion. However, the polarization of the liver cells and angiogenesis cells may vary from layer to layer. It should also be further developed and stabilized, as different results may appear depending on other auxiliary tools such as bioink.

Maher et al. successfully used a two-layer microfluidic system to culture liver cells [109]. They succeeded in the dynamic flow of a fluid and recreated ECM components based on the geometric properties of the liver, and they modeled the microbiology of liver sinusoidal waves by coculturing different liver cells. As a result of their study, hepatocytes that converted into cubic multicellular structures after several days of culturing were identified. Further, it was confirmed that the biliary tract was formed to maintain the cell pole. In addition, in a study by Ong et al., the use of microfluidic systems without fluid flow pumps succeeded in causing liver precursors to be efficiently differentiated into hepatocyte-like cells for long periods [110]. They used a microcolumn array method to arrange cells in 3D configurations and to enable a flow on both sides.

In addition, liver chips can be used to investigate in vitro interspecific drug toxicities. Animal experimental models are used as the main way to study the liver function and hepatotoxicity, but the method of evaluating DILIs with animal models has always been limited because the animal metabolic mechanisms are different from those of humans. Consequently, owing to false predictions based on animal experiments, many drugs have been withdrawn from the market. Therefore, the accurate identification of the specific toxicity of a species and identifying DILIs in human-relevant animals have become goals for the use of animal models [111]. Liver microchips were used to evaluate the mechanisms of action of various biomaterials and various liver damage phenotypes, such as bile stagnation and fibrosis markers. Based on these results, a cocultured liver microchip experiment successfully identified the hepatotoxicities of different species and their effects on all species.

However, there are limitations to these liver-chip models that should be eliminated in the design and fabrication of cell culture systems [112]. Complex systems need to be easily changeable, and the system throughput and cost efficiency are important parameters to consider when designing liver microsystems. This balance between operational simplicity and biological complexity can go a long way toward commercializing chips and increasing the affinity between biochips end users.

The fact that most current liver-chip models do not have a collection channel for bile outflows is a disadvantage, as bile can accumulate in the hepatocellular chamber, resulting in cytotoxicity. Therefore, the physiological host response to pathogens in the chip microenvironment must be studied appropriately.

### 3.3. Liver Studies Using Organoid or Spheroid Model

The pharmacokinetic processes of drugs in the human body include their absorption, distribution, metabolism, and exclusion. The liver is the major organ of drug metabolism and is an organ that greatly influences the pharmacological characteristics of drugs, such as bioavailability and wetting [113]. Having a lot of information on drug metabolism and safety can greatly raise the expectations for the discovery of new drugs and can guide the clinical use of drugs. Organoids are cell clusters formed in 3D through the self-organization of iPSCs in vitro as we already described [114]. They can reproduce the key functions of their own organs or tissues, and human iPSC-derived organoids have an especially great potential for disease modeling and drug testing along with long-term developmental research. When these organoids are produced with organoid-on-chips, they become a technology that combines organized organoids with organ-on-chips, therefore emerging as a new technology by helping to build a complete 3D organ model.

In particular, as the achievement of iPSC differentiation into hepatocytes, such as hepatocytes, bile duct cells, endothelial cells, and Kupffer cells, is known in many studies, studies in this field are making further progress [115]. iPSCs have the advantage of re-differentiating into mature cells, as they exhibit the expression of many transcription factors in embryonic stem cells [116]. However, despite this potential, the 2D cell cultures of iPSCs or primary cells are not sufficient for studying the replication of organ structures that mimic intercellular communication, the tissue microenvironment, or the in vivo environment. As a result, an organoid method, a 3D culture, has been developed to replicate complex cell–external matrix (ECM) and cell–cell interactions.

The first attempt at creating an in vitro liver structure using organoids was reported by the Michalopoulos group, which isolated adult rat liver cells and stored them in roller bottles coated with type I collagen in a medium containing dexamethasone, HGF, and EGF [117]. This proved that HGF and EGF are essential factors for the development of liver tissue and that dexamethasone is necessary for hepatocellular maturation, but the survival rate was very low, allowing them to survive only for a short period of time. To overcome this, a long-term culture method for self-renewable organoids was established. They exhibited self-regenerating properties in which, just by adding R-spondin1 to the culture medium, the cell expanded for a long time, acting as an adult conduit precursor cell, and maintained the ability to differentiate into hepatocytes [118]. In addition, mouse experiments have revealed vascularized liver organoids that produce mature liver cells by coculturing endoplasmic cells derived from iPSCs with hepatic lobe cells and human cord vein endothelial cells [118]. This demonstrates that the formation of functional vessels can trigger the maturation of iPSC-derived liver sprouts. Meanwhile, Asaiet et al. confirmed and reported that the factors of HGF, ANG, A2M, and PLG could induce the formation of liver organoids [119].

Guanet et al. formed a hepatocyte aggregate using iPSCs that were separated and cultured from Matrigel, and they generated organoids containing both hepatocytes and bile duct cells [120]. In other words, they cultured iPSCs in a medium containing BMP4 and FGF2 with inhibitors in the Wnt and PI3K pathways to induce endoderm differentiation and electric spheroids, and they were then recultured in a low-concentration Matrigel scaffold containing FGF10, OSM, and dexamethasone using a rotating ellipsoid [121].

Recently, studies testing tumor sensitivity to drugs using liver organoids have also been actively conducted. After establishing organoids of hepatocellular carcinoma, bile duct carcinoma, and hepatocellular-biliary carcinoma that replicate the structure and expression profile of the parent tumor, studies on drug testing and personalized medical care are also underway [122]. Meanwhile, a platform for drug testing using a liver-on-a-chip is also being actively produced. This allows liver organoids to be formed using perfusion microtubule chips, hepatocytes, and bile duct cells to measure the hepatotoxic effects of acetaminophen over time, allowing long-term liver-on-a-chip models as well as the liver to represent a new and likely platform for drug testing [101].

In addition to organoids, the liver spheroid system was initially developed using liver cell lines. The first paper describing the spheroids of human liver cells was published in 1993 using HepG2 cells [123]. However, their characterization and further development as models associated with the hepatotoxicity of drugs began with the use of spheroids almost 10 years later in 2013 as shown in a study by the Heinzle group [124]. They found better functionality and performance in spheroids compared to 2D cultures using bile-like and hepatocyte-like cells. Liver spheroids are formed from an ultralow-attachment plate as a drop of agglomeration or by using bioprinting to gather cells in one place inside the lid of the culture plate to induce the formation of the spheroids. Once formed, they are transferred to the selected culture plate format [125]. The spheroid formation of HepaRG and HepG2 cells can be improved through incubation in the liver biomatrix scaffolds produced in decellularized natural mice. The HepaRG cells used in the experiment showed the further enhancement of the spheroids, but a recent study using 150 compounds concluded that HepG2 spheroids are more sensitive to hepatotoxic drugs [126].

In addition to predicting the hepatotoxicity, human liver spheroids have also been used to investigate the toxicity mechanisms. Owing to increased metabolic activity in spheroids, many suggest that spheroids are more suitable for evaluating the toxicity caused by drug–drug interactions. For example, the proton pump inhibitor omeprazole, a known activator of the aryl hydrocarbon receptor (AhR), induced CYP1A2 activity in the spheroid, resulting in the increased metabolic activation and hepatotoxicity of dacarbazine, a CYP1A2 substrate [126]. In addition, it was able to identify genetic variants specific to various types of toxicity mechanisms by using spheroids, including amiodarone for mitochondrial toxicity, chlorpromazine for bile stagnation, and aflatoxin B1 for genetic toxicity [127].

Liver organoids can be composed of various liver cells; therefore, 3D cell culture systems have become much more informative than conventional 2D systems in studying interactions between cells where liver tissue damage occurs. As such, liver organoids or spheroids may be better suited for use in the experimental models of various liver diseases in order to represent the pathophysiological phenomena in liver tissues.

As we described in this manuscript, there have been advantages and disadvantages in the 3D culturing and application of liver cells. Table 3 summarizes and compares these pros and cons.

## 4. Conclusions

An in vitro study using hepatocytes may produce results similar to those in vivo, such as the reorganization of cell interactions, as long as conditions similar to the in vivo environment are maintained. However, considerable differences can be observed depending on the applied platform. New methods based on 3D culturing platforms composed of various biomaterials have been developed. Their ability to predict the drug metabolism, toxicity, and liver function represents a significant improvement compared to existing models that culture cells in 2D conditions. Three-dimensional culture technology enables the examination of liver functions to be directly related to living organisms and provides an opportunity to improve the liver-specific functions. Unlike 2D cell culture systems, 3D cell culture systems can be used in various forms depending on the structure of the extracellular substrates and culture containers, the cells’ properties, and the composition of the culture solutions.

However, despite the advances in the physiology and clinical applications that have been made, further research is still needed to develop and commercialize the 3D cell culture systems. In addition, the reproducibility of the models needs to be improved and standardized according to the laboratory conditions. Nevertheless, improved and standardized 3D cell culture systems are expected to have a significant ripple effect on medicine, new drug development, the bioindustry, and basic research.

## Figures and Tables

**Table 1:** Characteristic differences between two- and three-dimensional (2D and 3D) cell cultures.

	2D	3D	Reference
Cell morphology	Growing in a flat form on the floor.	Growing up in a 3D shape that can be found in tissues.	[5]
Cell–cell interaction	Cell-to-cell interactions are limited owing to the large proportion of interactions between the floor and the cell.	Cell-to-cell interactions are all connected and can form physiologically active conditions which are close to those of living organisms [6].	[6,7]
Cell differentiation and proliferation	Differentiation rate is not significantly improved, and proliferation shows a high growth rate.	Shows a very high cell differentiation rate compared to that of 2D cell cultures, and cell proliferation rates are achieved under conditions similar to the in vivo environment. Easy to control for rate.	[7]
Drug metabolism	Poor observation of drug metabolism.	The expression of various enzymes involved in drug metabolism has increased significantly, leading to a good drug metabolism process.	[8,9]
Drug sensitivity	Differences depending on the concentration of the drug; drug effects occur well.	High resistance to drugs compared to 2D cell cultures.	[9]

**Table 2:** Comparison of the basic characteristics of spheroids and organoids.

	Spheroids	Organoids	Reference
Sources	Can be formed through the tendency of aggregation of adhesive cells. It can be composed of several types of cells and can be formed by the cell’s own tissues.	Generated from both iPSCs and adult stem cells by mimicking the biochemical and physical cues of tissue development and homeostasis.	[49,50]
Applications	Spheroids can be useful for the discovery of disease-related cancers or drugs.	Organoids are useful for regenerative medicine, drug discovery, and disease modeling.	[51,52]
Considerations	It is economical and easy to manufacture, but it is difficult to cultivate for a long time.	Multiple cells can be combined into a single component and can closely imitate the actual organ, but a well-organized surrounding environment is needed.	[53]
3D culture conditions	Cultured with or without extracellular matrix and growth factors.	Needs extracellular substrates and growth factors	[49]

**Table 3:** Comprehensive comparison of hydrogel scaffolds, liver-on-chips, and liver organoids/spheroids.

In Vitro Application	Advantage	Disadvantage	Reference
Hydrogel 3D scaffold	It can be designed by calculating the 3D microenvironment and ECM characteristics. The expression of proteins and genes expressed in hydrogels is high, and the function and life of cells are improving with the development of technology.	Cell cultures are complicated due to the difference in materials, and the necrosis rate of cells occurring in the 3D cell culture model is a task to be overcome.	[128,129,130]
Liver-on-a-chip	3D cultures of liver tissues and cocultivation with other tissues are possible, and a fine environment suitable for spheroids can be manipulated in the laboratory. As a result, the expression rate of the CYP protein expressed in the reinforced liver is high, and a secondary structure can be formed. In addition, owing to the pattern created in the 3D space, the throughput is high, and the cost is relatively low.	The method of manipulating the chip is still complicated, and it is necessary to develop the supply system necessary for cell cultures on the chip. In addition, the growth of liver cells varies depending on the material of the chip. This is because standardized methods have not yet been established.	[131,132]
Liver Spheroid/ Liver Organoid	It can provide a microenvironment similar to a complex environment in vivo, enabling cell interactions, long incubation periods, and high expression of CYP 450 and transporters in the liver, and liver-specific functions are well maintained.	Owing to the limited size of the spheroids, it is still difficult to form large-sized spheroids. Additionally, cell properties may deform, or necrosis may occur in long-term cultures.	[101,132]

## Data Availability

Not applicable.
